# Effects of Genotypes and Treatment on Oxygenscan Parameters in Sickle Cell Disease

**DOI:** 10.3390/cells10040811

**Published:** 2021-04-05

**Authors:** Camille Boisson, Minke A. E. Rab, Elie Nader, Céline Renoux, Celeste Kanne, Jennifer Bos, Brigitte A. van Oirschot, Philippe Joly, Romain Fort, Alexandra Gauthier, Emeric Stauffer, Solene Poutrel, Kamila Kebaili, Giovanna Cannas, Nathalie Garnier, Cécile Renard, Olivier Hequet, Arnaud Hot, Yves Bertrand, Richard van Wijk, Vivien A. Sheehan, Eduard J. van Beers, Philippe Connes

**Affiliations:** 1 Laboratoire Interuniversitaire de Biologie de la Motricité (LIBM) EA7424, Team « Vascular Biology and Red Blood Cell », Université Claude Bernard Lyon 1, Université de Lyon, 69008 Lyon, France; camille.boisson2@gmail.com (C.B.); elie.nader@free.fr (E.N.); celine.renoux@chu-lyon.fr (C.R.); philippe.joly@chu-lyon.fr (P.J.); romain.fort@chu-lyon.fr (R.F.); alexandra.gauthier@ihope.fr (A.G.); emeric.stauffer@chu-lyon.fr (E.S.); solene.poutrel@chu-lyon.fr (S.P.); kamila.kebaili@ihope.fr (K.K.); 2 Laboratoire d’Excellence du Globule Rouge (Labex GR-Ex), PRES Sorbonne, 75006 Paris, France; 3 Laboratoire de Biochimie et de Biologie Moléculaire, UF de Biochimie des Pathologies Érythrocytaires, Centre de Biologie et de Pathologie Est, Hospices Civils de Lyon, 69500 Bron, France; 4 Central Diagnostic Laboratory—Research, University Medical Center Utrecht, Utrecht University, 85500, 3508 GA Utrecht, The Netherlands; m.a.e.rab@umcutrecht.nl (M.A.E.R.); j.f.bos-17@umcutrecht.nl (J.B.); b.oirschot@umcutrecht.nl (B.A.v.O.); r.vanwijk@umcutrecht.nl (R.v.W.); 5 Van Creveldkliniek, University Medical Center Utrecht, Utrecht University, 85500, 3508 GA Utrecht, The Netherlands; E.J.vanBeers-3@umcutrecht.nl; 6 Department of Pediatrics, Division of Hematology/Oncology, Baylor College of Medicine, Houston, TX 77030, USA; ckkanne@texaschildrens.org (C.K.); vivien.sheehan@emory.edu (V.A.S.); 7 Département de Médecine Interne, Hôpital Edouard Herriot, Hospices Civils de Lyon, 69008 Lyon, France; giovanna.cannas@chu-lyon.fr (G.C.); arnaud.hot@chu-lyon.fr (A.H.); 8 Institut d’Hématologie et d’Oncologie Pédiatrique, Hospices Civils de Lyon, 69008 Lyon, France; nathalie.garnier@ihope.fr (N.G.); cecile.renard@ihope.fr (C.R.); yves.bertrand@ihope.fr (Y.B.); 9 Centre de Médecine du Sommeil et des Maladies Respiratoires, Hôpital Croix Rousse, Hospices Civils de Lyon, 69004 Lyon, France; 10 Apheresis Unit, Etablissement Français du Sang Rhône Alpes, Centre Hospitalier Lyon Sud Pierre Bénite, 69310 Pierre Bénite, France; olivier.hequet@efs.sante.fr

**Keywords:** sickle cell disease, red blood cell deformability, oxygenscan, clinical severity, acute complication

## Abstract

(1) Background: The aim of the present study was to compare oxygen gradient ektacytometry parameters between sickle cell patients of different genotypes (SS, SC, and S/β^+^) or under different treatments (hydroxyurea or chronic red blood cell exchange). (2) Methods: Oxygen gradient ektacytometry was performed in 167 adults and children at steady state. In addition, five SS patients had oxygenscan measurements at steady state and during an acute complication requiring hospitalization. (3) Results: Red blood cell (RBC) deformability upon deoxygenation (EImin) and in normoxia (EImax) was increased, and the susceptibility of RBC to sickle upon deoxygenation was decreased in SC patients when compared to untreated SS patients older than 5 years old. SS patients under chronic red blood cell exchange had higher EImin and EImax and lower susceptibility of RBC to sickle upon deoxygenation compared to untreated SS patients, SS patients younger than 5 years old, and hydroxyurea-treated SS and SC patients. The susceptibility of RBC to sickle upon deoxygenation was increased in the five SS patients during acute complication compared to steady state, although the difference between steady state and acute complication was variable from one patient to another. (4) Conclusions: The present study demonstrates that oxygen gradient ektacytometry parameters are affected by sickle cell disease (SCD) genotype and treatment.

## 1. Introduction

Sickle cell disease (SCD) is a group of inherited red blood cell (RBC) disorders marked by the presence of a mutation in the β-globin gene leading to the production of an abnormal hemoglobin (Hb) [1]. The most common form of SCD is sickle cell anemia (SCA; HbSS disease (SS)). The mutation responsible for SCA occurs at the sixth position of the β-globin gene, leading to the replacement of a glutamic acid by valine, which causes the production of HbS. The main pathophysiological feature of HbS is that it polymerizes when deoxygenated, leading to the sickling of RBCs [2]. HbC is another Hb variant, also caused by a mutation at the sixth position of the β-globin gene, but in this case leading to the replacement of the glutamic acid by a lysine. HbC may form crystals under both oxygenated and deoxygenated conditions and promotes severe RBC dehydration through the activation of the K-Cl cotransporter [3,4]. The second most common form of SCD is HbSC disease (SC), in which RBCs from patients contain both HbS and HbC. Compared to SS patients, SC patients are generally considered to have a milder SCD phenotype. However, a significant proportion of these patients can develop frequent acute vaso-occlusive events and chronic organ damage [5,6]. 

The deformability of RBC from individuals with SCD is markedly abnormal, regardless of genotype. RBC deformability measured under the normoxic condition is decreased in both SS and SC patients, with SS patients showing the most pronounced decrease [7,8]. Several studies reported some associations between the degree of impairment of normoxic RBC deformability measured at steady state in SCD patients and the presence of chronic complications, such as priapism [9], leg ulcers [10], glomerulopathy [11], and retinopathy [5,12]. While RBC deformability measured in normoxic conditions has been found to be affected by vaso-occlusive crisis in SS [13], a recent study found no change in normoxic RBC deformability in SS patients between steady state and vaso-occlusive crisis [14]. Since HbS deoxygenation may further cause a decrease in RBC deformability, there is a need to fully assess the behavior of sickle RBC under deoxygenation to test whether the rheological changes could reflect clinical severity/complications. 

The recently developed technique of oxygen gradient ektacytometry allows for a more comprehensive functional characterization and rheological behavior of SCD RBCs over a range of oxygen tensions. We recently reported an association between oxygen gradient ektacytometry parameters measured at steady state and the frequency of vaso-occlusive crisis in both SS adults and children [15]. However, it remains unknown whether oxygen gradient ektacytometry parameters are modified during acute complications. Moreover, Rab et al. [16] recently reported on the rheological behavior of RBCs from three SC patients over an oxygen gradient. Despite the limited sample size, their findings suggest differences between SS and SC patients. However, it is unknown whether these differences apply to all subpopulations of SS patients, such as those with increased levels of fetal hemoglobin. Boisson et al. [17] also reported oxygen gradient ektacytometry data on 14 SC patients, but no comparison was performed with SS individuals. 

The aim of the present study was to compare oxygen gradient ektacytometry parameters between untreated and treated (hydroxyurea or chronic transfusion) SS and SC patients. In addition, we sought to determine if changes in deformability upon deoxygenation (EImin), deformability at normoxia (EImax), and point of sickling (PoS) correlated with onset and resolution of acute clinical complications in few patients. Comparison of SS and SC rheology biomarkers may also shed light on pathophysiological differences between the two genotypes.

## 2. Materials and Methods

### 2.1. Patients

A cohort of 167 adults and children at steady state and followed at the Hospitals of Lyon (France) was enrolled: 29 non-transfused and untreated (i.e., not on hydroxyurea therapy) SS patients older than 5 years old (SS), 11 non-transfused SS patients younger than 5 years old (SS < 5 years old), 45 SS patients treated with hydroxyurea (SS with HU), 54 SS patients treated with chronic red blood cell erythrapheresis (SS transfused), 22 SC (SC) patients, and 6 patients with S/β^+^-thalassemia (Sβ^+^). For the SS transfused group, blood was sampled just before erythrapheresis. It was decided to separate non-transfused SS patients younger than 5 years old from the other non-transfused (and without HU) SS patients in the analyses because it was previously demonstrated that RBC deformability measured in isotonic condition remained very high in SS patients younger than 5 years old because of the still high HbF level [7]. Information regarding the number of hospitalized patients with vaso-occlusive crises (VOC) and acute chest syndrome (ACS) in the previous two years and data on the presence of chronic complications, namely, leg ulcers, retinopathy, osteonecrosis, and glomerulopathy, were collected from the medical charts. Blood was collected in EDTA tubes for hematological and blood rheological measurements. In addition, five patients with SS had oxygenscan measurements at steady state and during an acute complication requiring hospitalization.

The study was conducted in accordance with the guidelines set by the Declaration of Helsinki and was approved by the Regional Ethics Committees (L14-127). Diagnosis of SS and SC was made according to expert recommendations [18]. SS or SC status was confirmed by DNA studies [19].

### 2.2. Hematological and Oxygenscan Parameters

Hb concentration, mean cell volume (MCV), mean corpuscular hemoglobin concentration (MCHC), and percentage of reticulocytes (Retic) were determined with a hematology analyzer (Advia, Siemens, Rungis, France). Lactate dehydrogenase levels (LDH) were determined by standard biochemical method. Blood viscosity was measured at native hematocrit (Hct), 25 °C, and at a shear rate of 90 s^−^^1^ using a cone/plate viscometer (Brookfield DVII+ with CPE40 spindle, Brookfield Engineering Labs, Natick, MA, USA). Ektacytometry was carried out with the laser-assisted optical rotational red cell analyzer (Lorrca, RR Mechatronics, The Netherlands) with the oxygenscan module to measure RBC deformability over an oxygen gradient. A volume of 50 µL of blood, standardized to a fixed RBC count of 200 × 10^6^, was mixed with 5 mL of high viscous (30 cP) Oxy-Iso polyvinylpyrrolidone (PVP) suspension [20]. The suspension was sheared at 30 Pa and 37 °C into the Couette system made of glass of the ektacytometer. The oxygen partial pressure (pO_2_) was gradually decreased from 160 mmHg to 20 mmHg (deoxygenation) and then re-increased to normoxic values [16,20,21]. The diffraction pattern was analyzed by the computer, and an elongation index (EI) that reflects RBC deformability was calculated. Several parameters can be derived: (1) EImax, the RBC deformability at normoxia; (2) EImin, the lowest RBC deformability reached upon deoxygenation; (3) point of sickling (PoS), the pO_2_ at which RBC deformability decreases below 5% of EImax during deoxygenation; and (4) delta EI, the difference between EImin and EImax. All measurements were standardized as recommended [20]. RBC aggregation was determined at 37 °C by the laser backscatter method, using the Lorrca MaxSis (RR Mechatronics, Hoorn, The Netherlands) after adjustment of the Hct to 40% with autologous plasma [22]. RBC aggregate strength was determined using a re-iteration procedure [22]: 7 separate pre-defined shear rates between 7.5 s^−1^ and 800 s^−1^ were applied on the RBC suspension, with or without alternating disaggregation shear rate, to locate the minimal shear rate needed to prevent RBC aggregation.

### 2.3. Statistics

A one-way analysis of variances with a post hoc Tukey test was used to compare the different parameters between the six populations at steady state. A paired student *t* test was used to compare the oxygenscan parameters in the same individuals between steady state and acute clinical complication. A Pearson’s test was used to test for the presence of correlations between biological parameters. Frequency of clinical complications was compared between SS and SC patients with a chi^2^ test. The significance level was defined as *p* < 0.05. Data are displayed as means ± SD. Statistical analyses were conducted using SPSS software (version 20, IBM SPSS Statistics, Chicago, IL, USA).

## 3. Results

### 3.1. Comparisons between Groups

The characteristics of the six groups of patients are shown in Table 1. Except for the SS < 5 years old group, the groups were not significantly different with respect to age. The fetal Hb (HbF) level was higher in the SS with HU group and tended to be higher in the SS < 5 years old (*p* < 0.10) group than in the SS group. These two groups also exhibited a higher HbF level than that of the SS transfused, SC, and Sβ^+^ groups. LDH levels of the three non-transfused SS subgroups were not significantly different. These groups had a higher LDH level than that of the transfused SS, SC, and Sβ^+^ groups. The transfused group had the lowest LDH values. Hb concentration was higher in the SS transfused, SC, and Sβ^+^ groups than in the SS group. The SS with HU group tended to have higher Hb than that of the SS group (*p* < 0.10), and the SC group had higher Hb than that of the SS transfused group. As expected, MCV was higher in the SS with HU group and lower in the SC and Sβ^+^ groups when compared to that of the SS group. MCHC was higher in the SC group and lower in the SS transfused and Sβ^+^ groups when compared to that of the SS group. The percentage of reticulocytes was lower in the SC and Sβ^+^ groups when compared to that of the four SS groups. RBC aggregation was slightly lower in the SC group than in the other groups, but no difference was observed for the RBC aggregate strength between the six groups.

Clinical characteristics were compared between the SS (i.e., SS patients without transfusion, without HU, and older than 5 years old) and SC groups (Table 2). The frequency of patients who exhibited at least one vaso-occlusive-like event (i.e., VOC or ACS) during the last two years was higher in SS than in SC individuals. In contrast, a higher proportion of SC patients had a retinopathy compared to SS patients. The proportion of osteonecrosis was not different between the two groups. The number of cases with glomerulopathy or leg ulcers was too low in these two groups to make a comparison.

**Figure 1:**
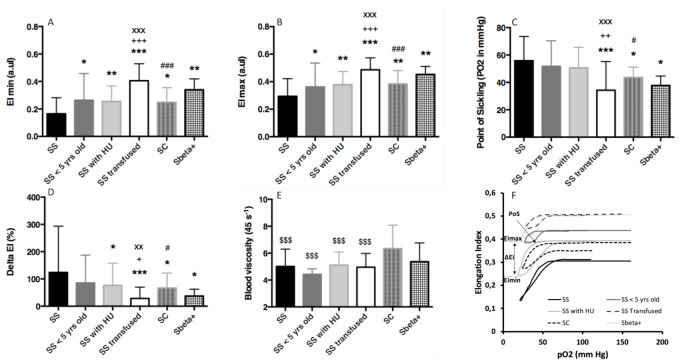
Comparisons of oxygen gradient ektacytometry parameters and blood viscosity between the 6 groups. (**A**) Minimum RBC deformability (Emin); (**B**) Maximum RBC deformability (EImax); (**C**) Point of Sickling (PoS); (**D**) Difference between EImin and EImax (Delta-EI); (**E**) Blood viscosity at 45 s^−1^; (**F**) Typical oxygenscan curves for the 6 populations tested. Compared to SS: * *p* < 0.05, ** *p* < 0.01, *** *p* < 0.001; different from SS < 5 years old: ^+^ *p* < 0.05, ^++^ *p* < 0.01, ^+++^ *p* < 0.001; different from SS with HU: ^xx^ *p* < 0.01, ^xxx^ *p* < 0.001; different from SS transfused: ^#^ *p* < 0.05, ^###^ *p* < 0.001; different from SC: ^$$$^ *p* < 0.001. (**F**) Typical oxygenscan curves for the six populations and the different key point of the curves: the RBC deformability at deoxygenation (EImin), the RBC deformability at normoxia (EImax), point of sickling (PoS) and the difference between EImin and EImax (delta EI).

The analysis of oxygenscan parameters (Figure 1) showed that EImin and EImax were lower in SS patients than in the five other groups. The SS transfused group also had higher EImin and EImax values than those of SS < 5 years old, SS with HU, and SC patients, but this group was not significantly different from those of the Sβ^+^ group. No difference was observed between the SC, SS < 5 years old, SS with HU, and Sβ^+^ groups for EImin and EImax. The PoS was not significantly different between the SS, SS < 5 years old, and SS with HU groups, and these three groups had higher a PoS than that of the SS transfused group. Both SC and Sβ^+^ patients had a lower PoS than that of the SS group. The delta EI was lower in the SS transfused group than in the SS, SS < 5 years old, SS with HU, and SC groups. The SC group had a lower delta EI than that of the SS group. Blood viscosity was higher in the SC group than in the four SS groups and tended to be greater in SC patients than in patients with Sβ^+^ (*p* < 0.10). Figure 1F shows typical oxygenscan curves for the six patient populations.

### 3.2. Associations between Oxygenscan and Hematological Parameters

**Figure 2:**
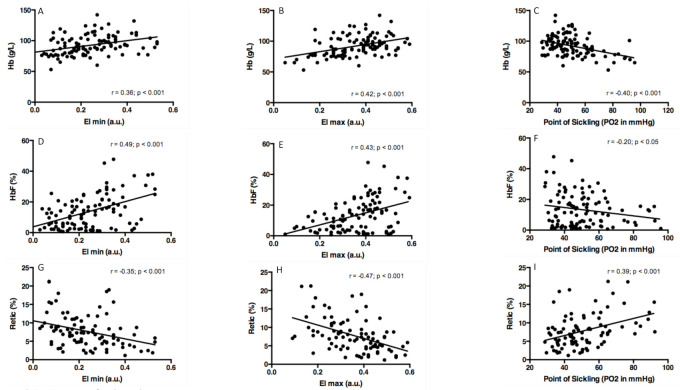
Correlations between oxygen gradient ektacytometry parameters and hemoglobin (Hb) concentration, fetal Hb (HbF) level and percent of reticulocytes (Retic) in all SS patients (excluding those under transfusion) and SC patients. (**A**) EImin vs. Hb; (**B**) EImax vs. Hb; (**C**) PoS vs. Hb; (**D**) EImin vs. HbF; (**E**) EImax vs. HbF; (**F**) PoS vs. HbF; (**G**) EImin vs Retic; (**H**) EImax vs. Retic; (**I**) PoS vs. Retic.

Correlations were tested between oxygenscan parameters and several hematological parameters in combined SS (i.e., non-transfused SS, without HU, and older than 5 years old) and SC patients. EImin (Figure 2A,D,G) and EImax (Figure 2B,E,H) were positively correlated with Hb concentration and HbF level, and negatively with the percentage of reticulocytes. We also observed a negative correlation between the PoS and, Hb (Figure 2C) and HbF levels (Figure 2F), and a positive correlation between PoS and the percentage of reticulocytes (Figure 2I). 

### 3.3. Oxygenscan Parameters and Acute Complication in SS

**Figure 3:**
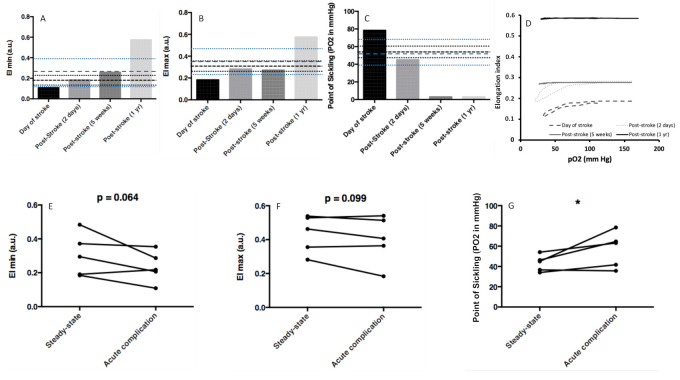
(**A**–**C**) Evolution of oxygen gradient ektacytometry parameters in a patient hospitalized for stroke event and then treated by chronic exchanged transfusion (5 weeks and 1 year after the event). (**A**) EImin; (**B**) EImax; (**C**) PoS;. A dashed bold black line indicates the mean, and thin black dashed lines indicate confidence intervals, calculated in the SS patients without any transfusion, older than 5 years old, not on hydroxyurea, and at steady state. A dashed bold blue line indicates the mean, and thin blue dashed lines indicate confidence intervals, calculated in the SS patients without any transfusion, younger than 5 years old, not on hydroxyurea, and at steady state. (**D**) Oxygenscan curves at the time of blood sampling in the patient who had a stroke event. (**E**–**G**) Comparisons of oxygenscan parameters between steady state and acute complication in 5 SS patients. (**E**) EImin; (**F**) EImax; (**G**) PoS. Significant difference: * *p* < 0.05.

Figure 3 shows the course of oxygen gradient ektacytometry parameters in SS patients during acute complications. This is illustrated by the case of a 3-year-old SS admitted to the hospital to determine if a stroke had occurred. EImin (Figure 3A) and EImax (Figure 3B) were very low, and the PoS (Figure 3C) was very high when compared to the mean values (and confidence intervals) determined in the non-transfused SS patients (both younger or older than 5 years old) of the present cohort. The computed tomography scan was negative, but the next day, an angio-IRM showed a new brain lesion (central gray nuclei, corona radiata, post and pre central gyrus, medial frontal gyrus) indicating stroke. While waiting for a blood transfusion, the patient was given IV fluids containing glucose. Blood was sampled before transfusion, and oxygen gradient ektacytometry parameters showed slight improvement compared to the day of hospital admission, possibly from IV hydration. After red cell exchange transfusion, EImin and EImax increased and PoS decreased to almost 0 mmHg. Figure 3D shows the different oxygen gradient ektacytometry curves and time of blood sampling.

Oxygen gradient ektacytometry measurements on five additional patients with acute complications (four VOCs and one stroke) are shown in Figure 3E–G). From a statistical point of view, we observed a trend towards a lower EImin (EImin decreased in four out of five patients) and EImax (EImax decreased in three out of 5fivepatients) and a significantly higher PoS (PoS increased in four out of five patients) during acute complication compared to steady state. 

## 4. Discussion

The higher EImin and EImax and lower PoS found in the SS patients receiving chronic red blood cell exchanges confirm previous findings [16] and clearly show that exchange blood transfusion allows the replacement of sickle RBCs by healthy RBCs with high deformability, hence decreasing the proportion of RBC containing HbS, which may polymerize upon deoxygenation. SS patients receiving HU treatment also had improved RBC deformability in both hypoxic and normoxic conditions compared to non-transfused SS patients, which reinforces previous findings showing increased normoxic RBC deformability in HU-treated individuals [23,24,25]. The same finding was observed for young SS children. This finding may be attributed to a higher HbF level observed in these two populations; HU stimulates HbF production, and young SS children (5 years old) may still have a high amount of HbF, because the switch from HbF to adult hemoglobin is delayed in SCD [26]. As previously demonstrated, the increase in the HbF level results in a decrease in the proportion of HbS inside the RBC and limits its ability to polymerize [27]. This is also supported by the correlations found between oxygenscan and hematological parameters (Figure 2). Although the slight difference in PoS between non-transfused SS individuals and young SS children or SS patients with HU patients did not reach statistical significance, we observed that deoxygenation had less impact on RBC deformability in the groups of patients with a high HbF level compared to when non-transfused SS patients (i.e., lower delta EI).

SC patients clearly exhibited better oxygenscan profiles than those of non-transfused SS individuals with higher RBC deformability in both normoxic and hypoxic conditions and lower PoS, which confirms the previous finding of Rab et al. [16] obtained in three SC patients. The comparisons of the oxygenscan parameters between SC patients and different SS subpopulations bring new findings that can be of clinical relevance. The oxygenscan characteristics of SC patients were very close to those observed in HU-treated patients and, to a lesser extent, to those observed in the youngest SS group. In addition, the difference in RBC deformability between hypoxic and normoxic condition was smaller in SC patients than in non-transfused SS patients. We speculate that RBCs from SC patients may pass from oxygenated vascular areas to microcirculatory territories where oxygen tension is severely decreased with better preservation of RBC deformability. This observation may explain why fewer patients from the SC group experienced VOC or ACS in the two previous years when compared to the SS group. The fact that the RBC response to deoxygenation in SC patients is less than that in SS patients may explain the lower frailty of RBCs in the former population, resulting in lower hemolysis and higher Hb, as is also the case for Sβ^+^ patients. 

Although the milder anemia in SC patients is beneficial for adequate oxygenation of the various organs in the body, the higher Hb concentration resulted in higher blood viscosity compared to the other population tested in the present study. Previous studies reported that both the milder anemia and the decreased normoxic RBC deformability found in SC patients are responsible for a rise in blood viscosity as compared to healthy individuals and SS patients [7,8]. The higher blood viscosity in SC patients is suspected to play a role in the progressive development of chronic disorders, such as retinopathy [5], which was present in half of the SC patients recruited in this study, while none of the non-transfused SS patients of similar age had retinopathy. 

Oxygen gradient ektacytometry parameters measured at steady state were recently demonstrated to be associated with clinical severity in SS adults and children [15], in particular with the frequency of vaso-occlusive crises. However, prior to the work described here, no study investigated the changes in oxygen gradient ektacytometry parameters during acute events, following patients longitudinally. The SS patients in our cohort who developed acute complications had a relatively low PoS at steady state compared to the values found in the rest of their group. During acute complications, the PoS rose in four of five patients studied longitudinally, with some patients reaching values above 60 mmHg. Since oxygen tension may vary between 60–65 mmHg and 30–40 mmHg in the pre-capillary arterioles depending on the tissue area (myocardium–brain vs. muscle area) [28], a PoS above 60 mmHg almost assures HbS polymerization if the transit time is long enough. The PoS of the patient admitted for stroke was very high (80 mmHg), suggesting that RBC may sickle in the pre-capillary arterioles of the brain where oxygen tension is high [29], which could then considerably impair blood flow in the microcirculation. This high PoS value could seem surprising, but Nash et al. [30] reported that RBC sickling may occur at an oxygen tension greater than 60 mmHg, and more recently, Lu et al. [31] reported a decrease in flow velocity of RBC from SS patients in a microfluidic device when oxygen tensions approached 70 mmHg, which was consistent with a rise in blood viscosity caused by the formation of sickled RBC. Chronic red blood cell exchange stabilized the patient clinically and allowed for normalization of oxygen gradient ektacytometry parameters with PoS values approaching 0 mmHg, thus reflecting the properties of normal RBCs, which do not show a loss of deformability upon deoxygenation. It is difficult to assess whether the differences in oxygen gradient ektacytometry parameters observed between steady state and the acute complications are the consequence of changing RBC properties (for instance, enhanced RBC oxidative stress, enhanced RBC dehydration, or accumulation of metabolites inside RBC) during clinical events or whether acute changes in the RBC population, detected by oxygenscan parameters, precipitate clinical complications—a classic chicken or the egg conundrum. Prospective studies and longitudinal follow-up of SCD patients are needed to answer this question. 

The present study also demonstrates that oxygen gradient ektacytometry parameters may not always reflect acute changes in the clinical status of SS patients since one patient had no change in EImin or PoS, and two patients had no change in EImax during acute events in comparison with steady state. A similar finding has been reported in a previous study where RBC deformability was measured in the normoxic condition in SS patients at steady state and during vaso-occlusive crisis [14]. The authors demonstrated that the mean RBC deformability of the SS group decreased during acute complication, but not all patients exhibited a decrease, and a few of them even showed an increase. This surprising finding may be explained by the fact that rigid sickled RBCs would be blocked or lysed in the microcirculation of some patients during vaso-occlusion and would therefore not be present in the blood samples. Further studies are needed to test the clinical usefulness of oxygen gradient ektacytometry parameters during acute clinical events in larger cohorts of SCD patients and to validate their predictive value.

## 5. Conclusions

The present study demonstrates that oxygenscan parameters are reflective of SCD genotype and treatment. In addition, the use of oxygenscan may have clinical application, as its parameters are associated with clinical severity and are sensitive to changes in the clinical status. Further studies are needed to validate its predictive values. 

## Figures and Tables

**Table 1:** Baseline hematological parameters and RBC aggregation in the 6 groups.

	SS (*N* = 29)	SS < 5 Years Old (*N* = 11)	SS with HU (*N* = 45)	SS Transfused (*N* = 54)	SC (*N* = 22)	Sb^+^ (*N* = 6)
Age (yrs)	24.1 ± 14.0	3.6 ± 1.2 ***	27.1 ± 13.0 ^+++^	23.4 ± 12.7 ^+++^	29.5 ± 17.2 ^+++^	21.2 ± 12.1 ^++^
HbF (%)	11.1 ± 12.4	15.7 ± 11.4	18.3 ± 8.5 ***	3.6 ± 3.7 ***^+++xxx^	4.6 ± 6.7 **^+++xxx^	6.8 ± 6.8 ^+xx^
HbC (%)	/	/	/	/	39.5 ± 11.2	/
HbA (%)	/	/	/	49.3 ± 16.2	/	14.3 ± 17.2
Hb (g/L)	84.7 ± 14.2	80.6 ± 14.7	90.4 ± 12.7 ^+^	96.1 ± 17.9 **^++x^	110.5 ± 13.9 ***^+++xxx###^	97.2 ± 24.3 *^+$^
MCV (fl)	78 ± 12	83 ± 11	95 ± 14 ***^++^	85 ± 10 **^xx^	74 ± 10 ^+x###^	62 ± 9 **^+++xxx###$^
MCHC (g/dL)	34.2 ± 1.7	34.1 ± 1.4	35.1 ± 1.3	32.9 ± 4.5 *^xxx^	35.6 ± 1.1 *^###^	31.2 ± 2.7 *^+xx$$^
LDH (IU/L)	380 ± 207	451 ± 240	334 ± 218	122 ± 210 ***^+++xxx^	224 ± 176 **^++x^	236 ± 209 *^+#^
Retic (%)	8.8 ± 3.7	10.4 ± 5.5	8.0 ± 4.2	10.1 ± 6.1 ^x^	4.1 ± 4.5 **^++xx###^	2.6 ± 1.9 **^++x##^
RBC aggregation (%)	60 ± 10	65 ± 9	64 ± 11	59 ± 11 ^x^	54 ± 10 *^++xxx#^	63 ± 5 ^$^
RBC aggregates strenght (s^−1^)	442 ± 246	550 ± 203	509 ± 272	424 ± 300	369 ± 236	445 ± 282

Different from SS: * *p* < 0.05, ** *p* < 0.01, *** *p* < 0.001; different from SS < 5 years old: ^+^ *p* < 0.05, ^++^ *p* < 0.01, ^+++^ *p* < 0.001; different from SS with hydroxyurea (HU): ^x^ *p* < 0.05, ^xx^ *p* < 0.01, ^xxx^ *p* < 0.001; different from SS transfused: ^#^ *p* < 0.05, ^##^ *p* < 0.01, ^###^ *p* < 0.001; different from SC: ^$^ *p* < 0.05, ^$$^ *p* < 0.01.

**Table 2:** Clinical complications in untreated SS and SC patients over 5 years of age.

	SS (*N* = 29)	SC (*N* = 22)
VOC or ACS (%)	63.3	37.9 *
Osteonecrosis (%)	16.7	20.7
Retinopathy (%)	0	47.0 ***

VOC = Vaso-occlusive crisis; ACS = Acute chest syndrome. Different from SS: * *p* < 0.05, *** *p* < 0.001.

## Data Availability

Data are available upon reasonable request to the corresponding author.
